# High prevalence of food insecurity, the adverse impact of COVID-19 in Brazilian favela

**DOI:** 10.1017/S1368980020005261

**Published:** 2020-12-28

**Authors:** Catarina V Manfrinato, Aluízio Marino, Vitória F Condé, Maria do Carmo P Franco, Elke Stedefeldt, Luciana Y Tomita

**Affiliations:** 1 Universidade Federal de São Paulo, Escola Paulista de Medicina, Department of Preventive Medicine, Rua Botucatu, 740, Vila Clementino, CEP 04023-062, São Paulo, Brazil; 2 Universidade Federal do ABC, São Paulo, Brazil; 3 Universidade Federal de São Paulo, Escola Paulista de Enfermagem, São Paulo, Brazil; 4 Universidade Federal de São Paulo, Escola Paulista de Medicina, Medicine Department, São Paulo, Brazil; 5 Instituto Estudos Avançados, Universidade de São Paulo, São Paulo, Brazil

**Keywords:** Food insecurity, COVID-19, Pandemic, Inequalities

## Abstract

**Objective:**

To investigate food insecurity (FI) prevalence in two favelas in Brazil in the early weeks of the social distancing policy, from 27 March 2020 to 1 June 2020.

**Design:**

A cross-sectional study using an online questionnaire to elicit information on socio-economic and demographic characteristics, the types of stores visited to buy food, and FI screening. The FI experience was evaluated according to the Brazilian Food Insecurity Scale. Factors associated with moderate or severe FI were investigated using the logistic regression model.

**Setting:**

São Paulo city, Brazil.

**Participants:**

Totally, 909 householders.

**Results:**

Eighty-eight per cent of the households included young women working as cleaners or kitchen assistants and in sales services. One-fifth of the participants were involved in the federal cash transfer programme, called *Bolsa Família.* There were 92 % households with children. The most frequent experience reported was uncertainty about food acquisition or receiving more (89 %), eating less than one should (64 %), not being able to eat healthy and nutritious food (46 %), and skipping a meal (39 %). Forty-seven per cent of the participants experienced moderate or severe FI. Factors associated with moderate and severe FI were low income, being a *Bolsa Família* recipient, having a low level of education and living in a household without children.

**Conclusions:**

Half of the participants experienced moderate or severe FI, and almost 10 % experienced hunger. Our data suggest that families with children were at a lower risk of moderate to severe FI. It is possible that nationally established social programmes such as *Bolsa Família* were protecting those families.

The coronavirus disease 2019 (COVID-19) pandemic has led to the tragic loss of human life with deep social and economic consequences, including those on food insecurity (FI). Disproportionate numbers of infections, hospitalisations and deaths from COVID-19 among already vulnerable communities have been observed^(1)^. The lack of regular access to nutritious and sufficient food experienced by such people puts them at greater risk of malnutrition, hidden hunger (i.e. micronutrient deficiencies), obesity and diet-related chronic diseases^(2,3)^.

According to the UN, FI is defined as ‘people having at all times, physical, social and economic access to sufficient, safe and nutritious food which meets their dietary needs and food preferences for an active and healthy life’^(4)^.

The impact of COVID-19 among Brazilian families could be significant. Brazil was experiencing FI before the coronavirus pandemic: more than half of adults were excess weight, and only 23 % reported recommended intake of vegetables and fruits, with a lower frequency observed among those with lower levels of education^(5)^. Among adolescents, a quarter was overweight and a third reported adequate fruit intake^(6–8)^.

The downward trend in moderate and severe FI from 2004 to 2013, observed in the Brazilian national survey, has been diverted in 2017/2018 reaching 12·7 %^(9)^. Higher prevalence of FI in 2017/2018 were observed among densely populated households, female householders and non-White individuals. Poor diet with lower amounts of vegetables, fruits, meat and milk among FI households was reported compared to food-secure households^(9)^. Among the poorest people, the prevalence of moderate and severe FI in Brazil ranged from 57·8 % to 75 % in 2011^(10,11)^.

In the COVID-19 pandemic, social distancing or quarantine is impossible in regions such as the Brazilian favelas that house approximately 13 million individuals, which are crowded and have limited access to clean water or hygiene supplies^(12)^. Those people work in informal or less flexible jobs and are at a higher risk of losing their jobs completely or partially.

On 25 February 2020, the first COVID-19 case was confirmed in São Paulo city, Brazil. Twenty days later, community transmission was announced. One month after the first COVID-19 case, a social distancing policy was adopted with the closing of schools and non-essential services.

Therefore, the objective of the present study was to investigate moderate and severe FI prevalence in two favelas in São Paulo city in the early weeks of the social distancing policy.

## Methods

A cross-sectional study was conducted in two favelas in São Paulo city, Brazil, between 27 March and 1 June 2020. The inclusion criterion was living in those communities. Only questionnaires answered by the householder or the person in charge of food acquisition and food intake patterns in the household were considered. We used a convenience sample based on a mobile phone number list from community partners, community leaders and local non-profit organisations.

The Heliopolis favela is the biggest and most densely populated shantytown located close to the downtown area of São Paulo city. The population size is not precisely known. According the last Brazilian census in 2010, the Brazilian Institute of Geography and Statistics (IBGE) estimated that 41 000 people lived in an area of approximately 1·2 km^2^ (50·6 % women, the mean of 3·4 dwellers per household). In an effort to obtain information about the COVID-19 infection, the IBGE estimated a population of around 61 000 or 15 200 households^(13)^. The UNAS, União de Núcleos, Associações dos Moradores de Heliopolis e Região, a local non-profit organisation, has estimated that 100 000 people live there. The UNAS was established in 1978 by a social movement in a squatter settlement in vacant lots^(14)^. The UNAS mission to transform Heliopolis was aimed at promoting citizenship and the community development. In 2019, UNAS was responsible for 52 social and educational projects benefitting around 10 000 people/month^(15)^. The authors have conducted educational and social actions with the non-profit organisation, including a nutritional educational programme with students from local public schools to stimulate a healthy eating habit.

Vila São José is a favela located in the south of São Paulo city. The last Brazilian census in 2010 estimated that 170 households in an area of approximately 134 km^2^ (51·2 % women, the mean of 4·1 dwellers per household). According to the IBGE 2020 estimate, the local population had about 353 households^(13)^. The community leader forwarded the questionnaire to 270 householders with high and very high vulnerability, which he had on his contact list. Respondents were asked to forward the questionnaire to other residents of Vila São José. On 24 May 2020, newspapers published reports that the peripheral regions of the city of São Paulo were where most people died from COVID-19^(16)^. The Vila São José community is located in a peripheral region. The researchers have conducted some social actions in Vila São José.

A sample of 245 household was sufficient to obtain a 95 % confidence level of proportion and a ±5 margin of error of if the observed prevalence of moderate and severe FI was 20 %, or the sample of 323 if prevalence was 30 %^(17)^.

### Data collection

A standardised online questionnaire was employed to elicit information on socio-economic and demographic characteristics, the frequency of food purchases, the types of stores visited to buy food, finances and FI screening after the social distancing policy came into effect. Data on the experience of FI were collected according to the Brazilian Food Insecurity Scale, which was adapted to ask about the time since the coronavirus outbreak^(18)^. In Heliopolis, the socio-economic and demographic characteristics questionnaire was administered on 27 March, and that about food access on 30 April 2020. In Vila São José, data were collected at one time from 1 May to 1 June 2020. We used the WhatsApp to send the survey invitation to individual contacts, and the Google Forms for the survey administration. The estimated time to answer these questions was approximately 20 min.

An online questionnaire was collected in the Heliopolis favela by the observatory ‘De Olho na Quebrada’ (‘Eyes in the Broken’). This UNA observatory is responsible for data assessment, and it has around 1200 families’ mobile phone numbers registered. To increase the observatory’s reach, Heliopolis is organised under four local coordinators responsible for reinforcing community participation in these questionnaires. Each questionnaire was available for 1 week. In Vila São José, the community leader forwarded the research to 270 householders with high and very high vulnerability that he had on his contact list. In both communities, respondents were asked to forward the questionnaire to other residents of the communities. Free Wi-Fi was available close to public school in Heliopolis. In Vila São José, households without Internet access were interviewed by a community leader.

The FI household level was assessed using the short version of the Brazilian Food Insecurity Scale (EBIA), adapted from the US Household Food Security Survey Measure (HPSSM) and validated for the Brazilian population^(18)^. The EBIA, validated in the 3-month reference period to capture seasonal associations, can be used for the most recent period of 1-month recall^(19)^. The EBIA items are ordered following the assumption, based on the qualitative research, that chronic FI results in a process managed at the household level, associated with a coping mechanism adapted to the severity of the FI challenge. Food-insecure households may first experience anxiety or worry after the loss of employment of the head of the household. If the situation does not improve, the household will start implementing strategies to extend food for longer periods, for example, adding water to milk or consuming more pasta and rice instead of vegetables and fruits. If FI then continues, household members will start reducing the amount of food to meet energy requirements, leading to hunger^(18)^. The EBIA short version is comprised of five questions and presents high sensibility and specificity compared to the original FI scale^(10)^. The householder should answer the question. A family that reported any experience of FI, by answering yes was scored with 1 point, and 0 points for no, for a maximum of 5 points in the questionnaire. The five questions were based on assessing the perception or experience of food intake in the household since the beginning of social distancing: (1) anxiety and worry about the ability to obtain food; (2) too poor to buy or receive more food; (3) whether the quality and variety of food have been compromised, including nutritious food; (4) quantity reduction and (5) skipping meals^(18)^. The total score classifications of the FI grade were (0) no insecurity, (1–2) mild insecurity, (3–4) moderate insecurity and (5) severe insecurity.

The income per person was converted from Brazilian Real to USD using the currency conversion rate during the period.

### Statistical analysis

Crude and relative distributions and median and interquartile ranges were calculated for the descriptive statistics. The Kruskal–Wallis test and chi-squared test were used to test differences between no FI or mild insecurity (EBIA ≤ 2) and moderate or severe FI (EBIA ≥ 3) in all measured correlates.

Factors associated with moderate or severe FI (EBIA ≥ 3) were investigated using the logistic regression model. The reference category for the exposure of interest was the lowest risk for FI. For income, the first quartile among households with no FI was the reference category. A level of significance of 5 % was adopted.

Statistical analysis was performed using STATA 14.0.

## Results

### Response rate

In the present study, 1172 participants answered the online survey. The participation rate was 922/1200 (77 %) in Heliopolis and 250/270 (93 %) in Vila São José. Of these, 123 (10 %) did not live in those communities, 86 (7·3 %) were not householders and for 54 (4·6 %), answers were repeated. Of these, 909 were considered eligible to participate; 697 lived in the Heliopolis favela and 212 in Vila São José.

### Demographic characteristics

The majority of the households included young women working as cleaners or kitchen assistants and in sales services. One-fifth were the *Bolsa Família* recipients, a federal cash transfer programme for individuals with extreme poverty, families with children who attend school and low-income pregnant women. There were many overcrowded households with children. Income information was not available for part of the study participants.

Only 52 (6 %) had graduated. Reported jobs were in education (56 %), administration (23 %), health care (10 %), human resources (6 %), law (4 %) and engineering (1 %).

### Food purchase

The majority of participants purchased food in a local supermarket (55 %) or a local market close to home (43 %), and only 2 % reported buying food in *feira*, an open outdoor public market that sells fresh vegetables and fruits. Majority observed an increase in food prices. Foods were purchased monthly (66 %), twice a month (23 %) and weekly (8 %).

### Food insecurity

More than half of the participants were in moderate and severe FI (56 %). The most frequent experience reported was uncertainty about food acquisition or receiving more, to eat less than one should, not being able to eat healthy and nutritious food, and skipping a meal. A quarter reported that food was consumed before buying or receiving more (Table 1).

**Table 1:** Brazilian´s favela household characteristics after social distancing to prevent COVID-19, Brazil, April, 2020 (*n* 909)

Brazilian´s favela	*n*	P50	%	P25, P75
Householder characteristics				
Female, %	796		88	
Age, years		33		28, 39
Monthly per capita income, USD	70·2		46·6	
*Bolsa família* recipients	236		26	
Occupation				
Cleaner, kitchen assistant and driver, deliveryman	369		40	
Salesman, freelance and autonomous	290		33	
Unemployed, housewife and retired	211		23	
Household characteristics				
Number of dwellers		4		3, 5
Household with child under 10 years	836		92	
Food insecurity				
Score at risk of food insecurity*		3		2, 4
No insecurity	47		5	
Mild insecurity	353		39	
Moderate insecurity	426		47	
Severe insecurity	83		9	
Uncertainty about food acquisition or receiving more	807		89	
Ran out of food before buying or receiving more	208		23	
Unable to eat healthy and nutritious food	421		46	
Ate less than one should	584		64	
Skipped a meal	350		39	

* No insecurity (0); mild insecurity (1–2 points); moderate insecurity (3–4 points); severe insecurity (5 points).

Participants not able to eat healthy and nutritious food reported lack of: milk (62 %); fruits and vegetables (18 %); rice, bean, and oil (14 %); and meat (1 %).

### Association with food insecurity

In the univariate analysis, moderate and severe FI was associated with a low level of education, occupation, income, presence of children (*P* < 0·001), living in Heliopolis (*P* = 0·001) and being a *Bolsa Família* recipient (*P* = 0·05). Being a *Bolsa Família* recipient and income were associated (*P* < 0·001).

In the multivariate model, factors associated with moderate and severe FI were low income, being a *Bolsa Família* recipient, low level of education and households without children (Table 2).

**Table 2:** Associated factors for moderate and severe food insecurity after social distancing from COVID-19, Brazil, April, 2020

Characteristics	Food-secure or mild food insecurity		Moderate or severe food insecurity			
	*n*	%	*n*	%	OR	95 % CI
Income per person, USD*						
≥62	223	62	200	48	1·00	
≤61	134	38	217	52	1·81	1·35, 2·41
*Bolsa família* recipient†						
No	309	77	364	72	1·00	
Yes	91	23	145	28	1·35	1·00, 1·83
Years of education						
≥9 years	221	56	203	40	1·00	
≤8 years	176	44	303	60	1·87	1·44, 2·44
Household with children						
Yes	383	96	453	89	1·00	
No	17	4	56	11	2·77	1·59, 4·76

* Total differs due to missing data.

† Federal cash transfer programme.

## Discussion

Our study examines the food access immediately following the COVID-19-related social distancing measure and school closure in two favelas in São Paulo city, Brazil. More than half of the participants experienced moderate or severe FI, and close to 10 % experienced hunger. Half were unable to eat healthy and nutritious food, while families with children were less likely to report moderate and severe FI. Almost all participants reported that food prices increased and the majority purchased food from supermarkets, but not from local outdoor markets where fresh vegetables and fruits were available.

### Limitations

The limitations of the present study should be considered. Socio-economic information and EBIA were assessed with a 1-month gap in Heliopolis. During this time, it is possible that more families lost unemployment, while others continued to be unemployed and suffered a deepened FI, so the magnitude of the association of income and FI could be underestimated. The web questionnaire was shared between both communities through local acting non-profit organisations and community leaders. It is possible that it reached many households due to their high capillarity and relevance to social policy. However, families that are going hungry could be under-represented since they may not have a mobile phone. No statistical probabilistic sample was drawn since the population size of the communities in question is unknown. The last Brazilian census was in 2010. The 2020 census was postponed due to the COVID-19 pandemic. Our results presented the sample of two favelas in São Paulo city with a different population size and localisation, in the downtown and periphery areas. However, they may not represent the current situation of the general population living in a shantytown; thus, the results cannot be generalised. However, given the uniqueness of the questions and our unawareness of any similar attempt in the vulnerable communities during the COVID-19 pandemic, the information can be of value to policy-makers.

### Food insecurity during COVID-19 pandemic

Early effects of the COVID-19 pandemic were also observed among low-income Americans: more than half of families experienced a decrease in income, and there was a 20 %–30 % increase in household FI since COVID-19, with 17 %–35·5 % new FI families^(20,21)^. FI were more likely among non-Hispanic, families with children and low educational level^(20,21)^. Among graduate and undergraduate students, 34·5 % were FI and were more likely to be non-White, younger, and overweight or obese^(22)^. Changes in food purchase have been observed with an increase in the amount of high-energy snack foods and desserts and sweets^(21)^.

According to the UN Department of Economic and Social Affairs (2020), the COVID-19 pandemic has affected all population segments, but it was reinforced among the poorest and most vulnerable people. Many of these people are workers in the informal economy^(23)^.

### Associated factors for food insecurity

Our data suggest that families with children were less likely to experience moderate and severe FI. It is possible that nationally established social programmes like *Bolsa Família* were protecting those families, as well as solidarity from non-profit organisations and private sectors that offer food kits, called *cesta básica* in Portuguese, comprised of rice and beans, pasta, salt, sugar, tomato pasta, beans, maize, maize flour, and vegetable oil and biscuits. To protect students from FI, the São Paulo state government offered an additional cash payment of USD 10 per month for *Bolsa Família* recipients.

### Food and nutrition security

The Brazilian public agenda has an intersectorial and participatory approach called food and nutrition security, which aims to develop public policies to warrant food and nutrition security: ensure human rights for a healthy, accessible and adequate diet, without compromising access to other essential needs, such as respecting healthy eating practices and cultural diversity, and should be socio-economically and agro-ecologically sustainable^(24)^. Through this programme, family farming from settlements should be supported in terms of sales to benefit socially vulnerable and marginalised populations^(25)^. In the present situation of poor access to healthy and fresh food in a highly vulnerable community, this programme should work^(26)^. However, in São Paulo city, the financing of social programmes has been reduced, including the family farming.

The present study showed the prevalence of moderate and severe FI after social distancing in two favelas in São Paulo city. More studies with representative sample conducted among low-income populations and vulnerable people are required. Our data suggest that encouraging family farming in close proximity of vulnerable communities, as well as implementing assistance programmes, especially for households without children, who are more likely to go hungry, can contribute to warranting food and nutrition security.
